# Infrared Brazed Joints of Ti_50_Ni_50_ Shape Memory Alloy and Ti-15-3 Alloy Using Two Ag-Based Fillers

**DOI:** 10.3390/ma12101603

**Published:** 2019-05-16

**Authors:** Chieh Lin, Ren-Kae Shiue, Shyi-Kaan Wu, Tsung-En Yang

**Affiliations:** 1 Department of Materials Science and Engineering, National Taiwan University, Taipei 106, Taiwan; f04527055@ntu.edu.tw (C.L.); rkshiue@ntu.edu.tw (R.-K.S.); 175075@mail.csc.com.tw (T.-E.Y.); 2 Department of Mechanical Engineering, National Taiwan University, Taipei 106, Taiwan

**Keywords:** Ti_50_Ni_50_ shape memory alloy, Ti-15-3 alloy, brazing, interfacial reaction, joint strength

## Abstract

The wettability, microstructures, and bonding strength of infrared brazing Ti-15-3 and Ti_50_Ni_50_ shape memory alloy using 72Ag-28Cu (wt.%) and 68.8Ag-26.7Cu-4.5Ti (wt.%) filler metals have been investigated. Only Ticusil^®^ active braze readily wets both Ti_50_Ni_50_ and Ti-15-3 substrates. Wetting of eutectic 72Ag-28Cu melt on Ti_50_Ni_50_ base metal is greatly ameliorated by adding 4.5 wt.% Ti into the alloy. The brazed Ti-15-3/BAg-8/Ti_50_Ni_50_ joint consists of Cu-Ti intermetallics in the Ag-rich matrix. The formation of interfacial Cu(Ti,V) and (Cu_x_Ni_1−x_)_2_Ti intermetallics next to Ti-15-3 and Ti_50_Ni_50_ substrates, respectively, is attributed to the wetting of both substrates. The brazed Ti-15-3/Ticusil^®^/Ti_50_Ni_50_ joint shows a vigorous reaction, which results in the formation of a large amount of Ti_2_Ni intermetallics in the joint. The maximum joint strengths using BAg-8 and Ticusil^®^ filler metals are 197 MPa and 230 MPa, respectively.

## 1. Introduction

Ti_50_Ni_50_ shape memory alloy (SMA) possesses excellent pseudoelasticity and shape memory effect [1,2]. Experimental results also demonstrate that Ti_50_Ni_50_ SMA is characterized by a very good biocompatibility [3,4,5]. Thus, it has led to extensive applications in biomedicine and engineering [6,7,8]. Titanium alloys have good specific strength as well as corrosion resistance, so they are widely applied in medical and aerospace applications. Ti-15-3 is a type of β-Ti alloy that has excellent forming characteristics at low temperatures [9]. Its chemical composition in wt.% is 3 Al, 3 Cr, 3 Sn, 15 V, and balance Ti. The strength of heat-treated Ti-15-3 primarily results from dispersed α-Ti precipitates in a β-Ti matrix [9]. 

Advancements in joining processes are crucial in many engineering alloy applications. Oliveira et al. updates recent advancements in joining Ti_50_Ni_50_ SMA [10]. Many studies on joining Ti_50_Ni_50_ and its effects on the joint performance have been evaluated [11,12,13,14,15]. Additionally, dissimilar joining of Ti_50_Ni_50_ SMA is a complex issue relating to the joined base metal because of brittle intermetallics formation in the joint [10]. Joining Ti_50_Ni_50_ to Ti-15-3 alloy is of great interest because it provides excellent combined functional characteristics owned by Ti_50_Ni_50_ and the mechanical/corrosion features of Ti-15-3 [10]. 

It is well known that brazing is more appropriate than welding for bonding dissimilar alloys. Infrared brazing is more suitable than furnace brazing for evaluating the evolution of joint microstructures because of its heating rate as high as 50 °C/s [16,17]. The base metal is less affected by the thermal history of infrared brazing, so it is applied in the experiment. It is important to use an appropriate filler foil for brazing Ti_50_Ni_50_ SMA and Ti-15-3 alloy. The wettability of braze melt on the base metal and reaction(s) at the interface between the braze melt and base metal must both be considered in filler metal selection. According to previous studies, many titanium alloys are well brazed with Ag-based braze alloys [18,19,20,21]. However, the Ag-Cu eutectic melt cannot effectively wet the Ti_50_Ni_50_ substrate unless the active ingredient, Ti, is alloyed into the braze [22]. Ti_50_Ni_50_ SMA and 316 stainless steel have been infrared brazed using the Ticusil^®^ (68Ag-26.7Cu-4.5Ti, wt.%) braze alloy, and the highest shear strength of such joints is 237 MPa for specimens joined below 950 °C [20]. 

In this study, BAg-8 (72Ag-28Cu, wt.%) and Ticusil^®^ foils were selected as the brazing fillers. The eutectic of the BAg-8 filler is 780 °C, and the liquidus of the Ticusil^®^ filler is 850 °C. This research was focused on wetting and infrared brazing of Ti_50_Ni_50_ and Ti-15-3 alloys. Microstructures, dynamic wetting behaviors of the two fillers, as well as bonding strengths of joints were examined.

## 2. Materials and Methods 

Ti_50_Ni_50_ SMA was made by using a vacuum arc remelter with pure titanium and nickel pellets. Pure titanium and nickel granules were degreased with 1HF-5HNO_3_-64H_2_O (in cm^3^) and 2HCl-25HNO_3_-75CH_3_COOOH (in cm^3^) solutions before being vacuum arc remelted. During the melting, the master alloy was melted at least six times with a pure titanium block as the getter, and its weight loss was below 0.1%. The Ti_50_Ni_50_ ingot was hot-rolled and homogenized at 900 °C to obtain plates of 3 mm thickness. A Ti-15-3 plate with a thickness of 3 mm was used as the substrate. BAg-8 and Ticusil^®^ fillers with 50 μm in thickness were obtained from Morgan Advanced Materials Inc., Berkshire, UK.

**Figure 1:**
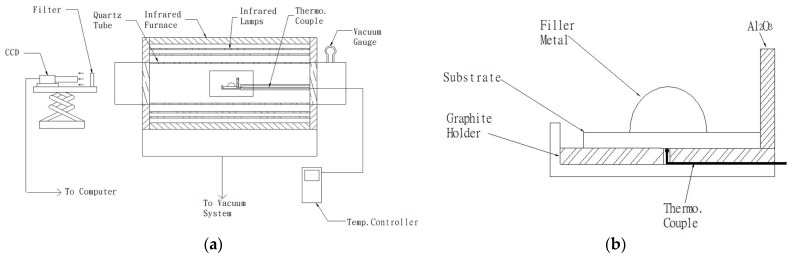
(**a**) Experimental installation of the wetting angle test and (**b**) thermocouple location of the specimen holder in part (**a**).

Figure 1a displays the experimental installation of the wetting angle test, and it contains infrared lamps, a sample holder, and a CCD recording system [23]. The infrared furnace (SINKO-RIKO RHL-816C, ULVAC, Tokyo, Japan) under a vacuum of 5 × 10^−8^ bar was used in the wetting angle and brazing experiments. Infrared furnace heating rate was fixed at 10 °C/s during the test. The braze alloy prepared by a vacuum arc remelting furnace with a near spherical shape, weighing 0.15 g was applied in the wetting angle experiment. Figure 1b shows the thermocouple location of the specimen holder, and the thermocouple was in contact with the specimen holder [23]. 

All joined surfaces were ground sequentially with SiC papers of 240, 400, 600, 800, and 1200 grit with a scratch depth of approximately 15 μm [24]. Next, they were cleaned in an ultrasonic bath prior to brazing. The power of the ultrasonic bath was 150 W with a fixed frequency of 43 kHz. The brazing foil was placed in between the Ti_50_Ni_50_ and Ti-15-3 base metals. The specimen was enclosed by a graphite fixture with a thermocouple inserted into the lower graphite plate (Figure 1b). Preheating temperatures of BAg-8 and Ticusil^®^ brazes were 700 and 800 °C for 300 s, respectively. Table 1 shows the wetting angle and shear test conditions applied in tests.

**Figure 2:**
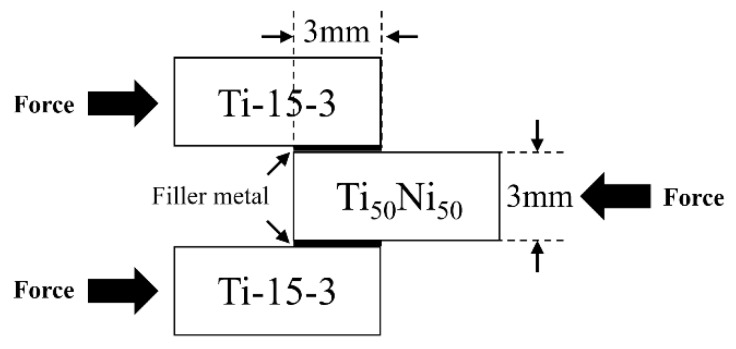
Illustration of the shear test sample and applied forces in shear test.

Bonding strengths of joints were assessed with the symmetric double lap shear tests as described in previous reports [11,18,19]. Figure 2 displays the shear test sample and applied forces in the shear test. The brazing filler foil, indicated by bold lines with the width of 3 mm, was placed in between the Ti_50_TNi_50_ and Ti-15-3 base metals. Shear tests of the infrared brazed joint were evaluated using a Shimadzu AG-10 universal testing machine (Shimadzu Corporation, Nakagyo-ku, Japan) and its crosshead speed was kept at 0.0167 mm/s. The cross-sections of the infrared brazed joint were inspected with a Nova Nano 450 field emission scanning electron microscope (FESEM, FEI Corp., Hillsboro, OR, USA). Operation voltage of 15 kV and 1 μm spot size were applied in the inspection.

## 3. Results and Discussion

### 3.1. Dynamic Wetting Angle Test

**Figure 3:**
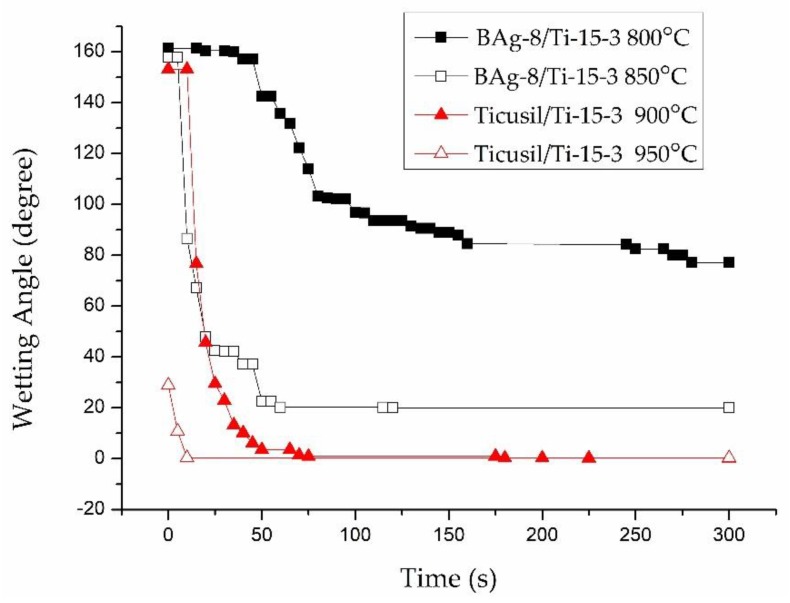
Wetting angles of BAg-8 and Ticusil^®^ on the Ti-15-3 substrate at different temperatures.

Figure 3 illustrates the dynamic wetting behaviors of the BAg-8 and Ticusil^®^ filler metals on Ti-15-3 substrate at various temperatures for 300 s. From Figure 3, one can find that the BAg-8 braze could not well wet the Ti-15-3 substrate at 800 °C, but the wettability was greatly increased by increasing the testing temperature to 850 °C, at which the wetting angle of 20° was readily reached after 60 s. On the other hand, the molten BAg-8 braze demonstrated poor wettability on a Ti_50_Ni_50_ substrate, even when the testing temperature was increased to 850 °C [22]. Figure 3 also shows the dynamic wetting angle behavior of the Ticusil^®^ alloy on Ti-15-3 base metal at 900 and 950 °C. The molten Ticusil^®^ braze effectively wetted the Ti-15-3 substrate, and the wettability was slightly improved by increasing the test temperature from 900 °C to 950 °C. The wetting angle approached zero after 70 s in both test conditions. Unlike the BAg-8 filler metal, the Ticusil^®^ alloyed with 4.5 wt.% Ti active ingredient, which greatly ameliorates wetting on both Ti-15-3 and Ti_50_Ni_50_ substrates [21].

**Figure 4:**
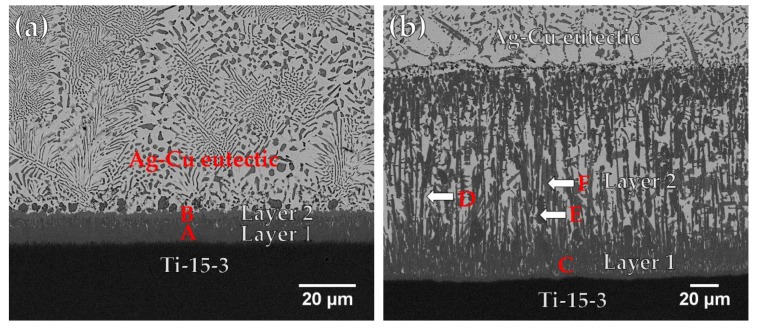
Cross-sectional observations of FESEM BEIs of Ti-15-3 substrate after wetting test using BAg-8 filler metal at (**a**) 800 and (**b**) 850 °C for 300 s.

Figure 4 and Table 2 display FESEM backscattered electron images (BEIs) and chemical analysis results for the BAg-8 filler metal after wetting angle tests of the Ti-15-3 substrate at 800 and 850 °C for 300 s, respectively. In Figure 4a, the microstructures are primarily the Ag-Cu eutectic and two gray interfacial reaction layers. The darker layer 1 near the Ti-15-3 substrate, marked A, is Cu(Ti,V) intermetallics with minor white acicular Ag-rich phase, and the lighter layer 2 near the Ag-Cu eutectic phase, marked B, is Cu_4_Ti. The thickness of layer 1 is about 10 μm, and that of layer 2 is less than 5 μm. The interfacial reaction layers are greatly coarsened, and their compositions were changed by increasing the test temperature from 800 to 850 °C. In Figure 4b, two interfacial reaction layers can be seen. Layer 1 is mainly composed of Cu(Ti,V) intermetallic compound, marked C, mixed with a minor acicular Ag-rich phase. Layer 2 is composed of three phases: the white Ag-rich phase, marked D; the darker gray Cu_4_(Ti,V)_3_ intermetallics, marked E; and the lighter gray Cu_4_Ti intermetallics, marked F. The thicknesses of layer 1 and layer 2 are 25 μm and 120 μm, respectively. Based on the Ti-V phase diagram, β-Ti and V are completely soluable with each other at high temperature [25]. Therefore, the Cu-Ti interfacial reaction layers alloyed with a little V are presented as Cu(Ti,V) and Cu_4_(Ti,V)_3_ compounds. However, no interfacial reaction layer was observed from the Ti_50_Ni_50_ side [26]. Fine Ag-Cu eutectic dominates the entire cross-section. The lack of an interfacial reaction layer results in insufficient wetting of the BAg-8 melt on the Ti_50_Ni_50_ base metal, as described in the previous work [22]. 

**Figure 5:**
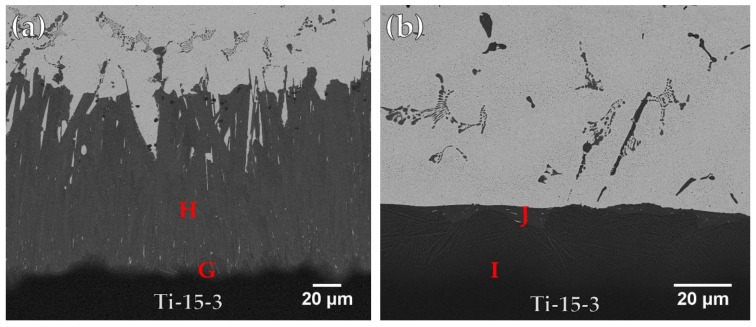
Cross-sectional observations of FESEM BEIs of Ti-15-3 substrate after wetting angle test using Ticusil^®^ filler metal at (**a**) 900 and (**b**) 950 °C for 300 s.

Figure 5 and Table 3 present FESEM BEIs and chemical analysis results using the Ticusil^®^ filler metal after wetting angle tests of Ti-15-3 base metal at 900 and 950 °C for 300 s, respectively, in which interfacial reaction layers between the braze and base metal are found. In Figure 5a, it can be seen that the Cu content in the braze melt reacted vigorously with Ti and formed a gray Ti-Cu-V reaction layer (marked G) next to the Ti-15-3 substrate. A very thick CuTi intermetallic layer (marked H) is also visible. In Figure 5b, the thickness of the CuTi reaction layer was decreased due to better fluidity of the braze melt at increased brazing temperature. Most of the molten melt overflowed out of the joint without reacting with the Ti-15-3 substrate. 

### 3.2. Microstructures of Brazed Joints

#### 3.2.1. Ti-15-3/BAg-8/Ti_50_Ni_50_ Joints

**Figure 6:**
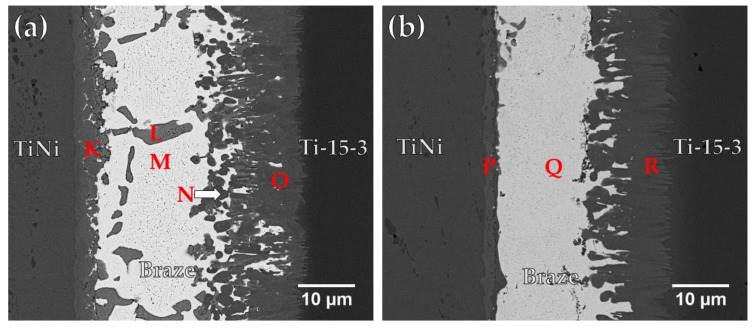
FESEM BEI observations of Ti_50_Ni_50_/ BAg-8/ Ti-15-3 joints brazed at (**a**) 800 and (**b**) 850 °C for 300 s.

Figure 6a and Table 4 display the FESEM BEI and EDS chemical analysis results of a Ti-15-3/BAg-8/Ti_50_Ni_50_ specimen infrared brazed at 800 °C for 300 s. In Figure 6a, the infrared brazed joint mainly consists of Cu_2_Ti (marked L) and Ag-rich matrix (marked M). There are two interfacial reaction layers in the brazed joint. The interfacial layer between the braze alloy and Ti-15-3 is a Cu(Ti,V) compound, marked O, and that between the braze alloy and Ti_50_Ni_50_ is a Cu-Ni-Ti compound, marked K [27]. The Cu-Ni-Ti phase is expressed by (Cu_x_Ni_1−x_)_2_Ti, where x is between 0.23 and 0.75 at % [27]. It is obvious that the reaction between Cu and Ti plays an important role in brazing two Ti-based alloys. The interfacial Cu-Ni-Ti reaction layer results from the reaction between the Cu-Ti rich liquid and the Ti_50_Ni_50_ base metal. 

Figure 6b displays FESEM BEI and EDS chemical analysis results of a Ti-15-3/BAg-8/Ti_50_Ni_50_ specimen infrared brazed at 850 °C for 300 s. Increasing the brazing temperature enhances the interfacial reaction among the braze melt and two substrates. Most of Cu in the molten braze readily react with Ti_50_Ni_50_ and Ti-15-3 substrates to form interfacial (Cu_x_Ni_1−x_)_2_Ti and Cu(Ti,V) reaction layers. According to Figure 6b, the Cu content in the molten BAg-8 braze was almost completely consumed. No Ag-Cu eutectic is visible in the figure, and only the white Ag-rich matrix, marked Q, remains in the center of the brazed joint. Meanwhile, the Cu_2_Ti phase disappears from the brazed joint at higher brazing temperature due to the enhanced interfacial reactions which consume Cu in the braze melt. 

#### 3.2.2. Ti-15-3/Ticusil^®^/Ti_50_Ni_50_ Joints

**Figure 7:**
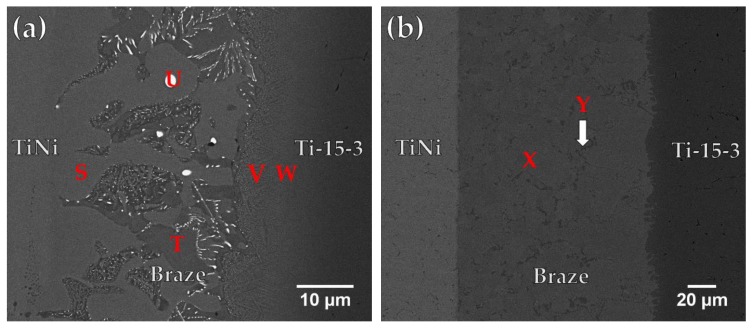
FESEM BEI observations of Ti_50_Ni_50_/ Ticusil^®^/ Ti-15-3 joints brazed at (**a**) 900 and (**b**) 950 °C for 300 s.

Figure 7a and Table 5 show FESEM BEI and chemical analysis results of a Ti-15-3/Ticusil^®^/Ti_50_Ni_50_ sample brazed at 900 °C for 300 s. The joint is mainly composed of CuNiTi (marked S) and CuTi_2_ (marked T) with a few Ag-rich particles (marked U). Two interfacial reaction layers were identified from the EDS analyses. A continuous CuTi_2_ interfacial layer (marked V and W) can be observed between the braze and Ti-15-3 substrate, and no reaction layer exists between the braze and Ti_50_Ni_50_ substrate. The CuNiTi phase belongs to the category of SMA, so it is regarded as beneficial to the shape memory effect of the brazed joint [1]. 

Figure 7b displays FESEM BEI observation of Ti_50_Ni_50_/ Ticusil^®^/ Ti-15-3 joint brazing at 950 °C for 300 s. It is worth mentioning that most of the braze melt overflowed out of the joint at 950 °C. Increasing the brazing temperature to 950 °C, dissolution of Ni and Ti ingredients from Ti_50_Ni_50_ and Ti-15-3 substrates into the braze melt enhances and forms the Ti_2_Ni intermetallic compound in the central brazed zone. According to Figure 7b and Table 5, the central brazed region is filled with Ti_2_Ni intermetallic compound (marked X) and the Ti-rich phase (marked Y). Increasing the brazing temperature from 900 to 950 °C causes partial loss of Ticusil^®^ braze melt from the joint and forms Ti_2_Ni intermetallic compound in the central region of the joint due to dissolution of two base metals into braze melt. 

### 3.3. Shear Strengths of Brazed Joints

**Figure 8:**
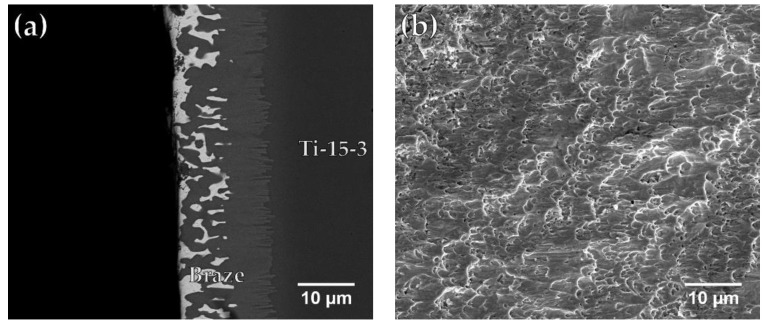
FESEM (**a**) BEI cross section and (**b**) SEI fractograph of Ti-15-3/BAg-8/Ti_50_Ni_50_ joint brazed at 850 °C for 300 s after shear test.

**Figure 9:**
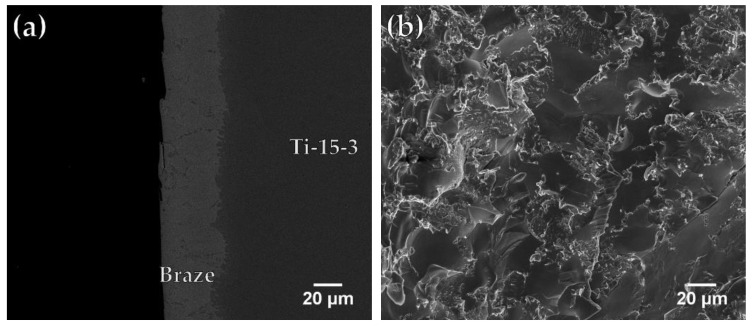
FESEM (**a**) BEI cross section and (**b**) SEI fractograph of Ti-15-3/Ticusil^®^/Ti_50_Ni_50_ joint brazed at 950 °C for 300 s after shear test.

Table 6 displays the average shear strengths of joints fabricated under various brazing conditions. For the BAg-8 filler, shear strength of the joint is increased by increasing the brazing temperature because of the better wettability of the filler at higher temperature, as illustrated in Figure 3. For the Ticusil^®^ filler, the shear strength of the brazed joints was also slightly higher for the joints brazed at higher temperature, although the Ticusil^®^ filler has quite good wettability at both 900 °C and 950 °C. The best strength of the BAg-8 joints brazed at 850 °C for 300 s was 197 MPa while that of the Ticusil^®^ joints brazed at 950 °C for 300 s was 230 MP. Cross-section BEIs and SEI (secondary electron image) fractographs of the fractured joints are shown in Figure 8 and Figure 9, respectively.

Figure 8a,b present an SEM BEI cross-section and a SEI fractograph of the fractured Ti-15-3/BAg-8/ Ti_50_Ni_50_ joint brazed at 850 °C for 300 s. The brazed specimen in Figure 8a fractured between the interfacial Cu(Ti,V) reaction layer and the Ag-rich matrix. The fractograph shown in Figure 8b presents quasi-cleavage fracture, and the morphology of fractured surface is mixed with ductile dimples and brittle facets. Figure 9a,b show an FESEM BEI cross-section and an SEI fractograph of the joint brazed at 950 °C for 300 s. The fracture location shown in Figure 9a is along the central brittle Ti_2_Ni intermetallic compound. The existence of central brittle intermetallics in the joint results in brittle cleavage fracture of the entire SEI fractograph as illustrated in Figure 9b. 

Appropriate wetting ability of the braze melt on the base metal is one of the prerequisites to acquire a good joint. Alloying 4.5 wt.% Ti into the Ag-Cu eutectic, e.g., Ticusil^®^ filler, improves its wetting on both substrates. However, alloying Ti into BAg-8 alloy increases both liquidus and brazing temperatures, which results in enhanced interfacial reaction and dissolution of both substrates. The amount of intermetallic phase in the brazed zone is significantly increased. For the Ti-15-3/Ticusil^®^/Ti_50_Ni_50_ joint, brazing at 950 °C for 300 s presents the best bonding strength of 230 MPa due to the presence of Ti_2_Ni matrix in the joint. A brittle, cleavage-dominated fractograph is observed after the shear test. In contrast, failure of interfacial reaction layers and ductile Ag-rich phase are found after the shear test of the BAg-8 brazed joint. Its average shear strength is slightly decreased to 197 MPa. The morphology of the fractured surface is mixed with ductile dimples and brittle facets. 

Dissimilar infrared brazing of Ti-15-3 and Ti_50_Ni_50_ shape memory alloy using BAg-8 and Ticusil^®^ foils have been evaluated. BAg-8 and Ticusil^®^ fillers can effectively braze Ti_50_Ni_50_ shape memory alloy and Ti-15-3. The dissimilar infrared brazed joints show moderate average shear strengths of between 172 and 230 MPa. Dissimilar infrared brazing is faster than the traditional one. Additionally, very limited distortion of the joint was observed. It demonstrates the potential for application, e.g., the bimetal product with complex geometry which is not suitable for the cladding process. 

## 4. Conclusions

Dissimilar infrared brazing of Ti_50_Ni_50_ SMA and Ti-15-3 β-Ti alloy with BAg-8 and Ticusil^®^ foils have been investigated. Important conclusions are listed below.

1. BAg-8 and Ticusil^®^ brazing foils wet the Ti-15-3 substrate. The wettability of Ag-Cu eutectic is greatly ameliorated by alloying with 4.5 wt.% Ti.
2. A Ti-15-3/BAg-8/Ti_50_Ni_50_ joint brazed at 800 °C for 300 s consists of Cu_2_Ti intermetallics in the Ag-rich matrix, and only the Ag-rich matrix remains in a joint infrared brazed at 850 °C for 300 s. An interfacial Cu(Ti,V) reaction layer next to Ti-15-3 and interfacial (Cu_x_Ni_1−x_)_2_Ti phase next to the Ti_50_Ni_50_ are attributed to the reactive wetting of both substrates.
3. A Ti-15-3/Ticusil^®^/Ti_50_Ni_50_ joint brazed at 900 °C for 300 s consists of CuNiTi and CuTi_2_. A CuTi_2_ interfacial layer is next to the Ti-15-3 substrate. For the sample, brazing at 950 °C causes all Ag-rich melt to overflow out of the specimen, leaving the Ti_2_Ni matrix and Ti-rich particles in the joint.
4. The best joint strengths using BAg-8 foil and Ticusil^®^ are 197 and 230 MPa, respectively. For the BAg-8 joint, cracks propagate at the location between the interfacial Cu(Ti,V) and the Ag-rich phase. In a Ticusil^®^ brazed joint, cracks are initiated and propagate along the central Ti_2_Ni intermetallic compound in the brazed zone.
5. The dissimilar infrared brazed joints show moderate average shear strengths of between 172 and 230 MPa. It is a much faster process than that of the traditional furnace brazing, and very limited distortion of the joint is observed. It shows potential for future application in industry.

## Figures and Tables

**Table 1:** Wetting angle and shear test conditions applied in test.

Filler Foil	Substrate	Brazing Period	Brazing Temperature			
			800 °C	850 °C	900 °C	950 °C
BAg-8	Ti-15-3	300 s	W	W		
Ticusil^®^	Ti-15-3	300 s			W	W
BAg-8	Ti_50_Ni_50_, Ti-15-3	300 s	S/M	S/M		
Ticusil^®^	Ti_50_Ni_50_, Ti-15-3	300 s			S/M	S/M

W: Wetting angle sample; M: Metallographic sample; S: Shear test sample.

**Table 2:** Chemical analyses in Figure 4.

at %	A	B	C	D	E	F
Ag	1.8	1.3	2.2	86.8	2.6	1.5
Al	1.1	0.2	1.7	-	0.1	-
Cr	1.8	-	0.9	-	2.6	0.4
Cu	47.1	78.8	43.0	13.1	53.3	78.2
Sn	0.3	-	0.6	-	0.3	0.1
Ti	41.9	18.9	43.6	0.2	32.6	19.9
V	5.9	0.7	7.9	-	8.5	-
phase	Cu(Ti,V)	Cu_4_Ti	Cu(Ti,V)	Ag-rich	Cu_4_(Ti,V)_3_	Cu_4_Ti

**Table 3:** Chemical analyses in Figure 5.

at %	G	H	I	J
Ag	2.9	3.9	2.1	4.9
Al	0.1	1.2	3.7	2.3
Cr	4.0	0.5	3.2	0.5
Cu	17.4	45.3	15.2	43.5
Sn	1.6	0.2	1.3	0.2
Ti	56.6	43.1	58.9	44.3
V	17.4	5.9	15.6	4.3
phase	Ti-Cu-V	CuTi	Ti-Cu-V	CuTi

**Table 4:** Chemical analyses in Figure 6.

at %	K	L	M	N	O	P	Q	R
Ag	0.8	2.0	87.5	1.9	2.1	1.2	87.2	3.5
Al	0.3	7.7	0.1	1.2	1.3	0.6	-	1.5
Cr	0.2	-	1.8	1.0	1.4	-	1.3	1.1
Cu	58.1	65.2	8.4	37.5	43.9	51.6	10.1	46.1
Ni	10.6	0.8	1.3	0.6	-	15.6	0.9	-
Sn	-	0.3	-	1.8	0.9	0.1	-	0.2
Ti	29.8	23.3	0.4	46.0	41.1	30.4	0.5	43.0
V	0.1	0.6	0.6	10.0	9.2	0.5	-	4.7
phase	(Cu_x_Ni_1−x_)_2_Ti	Cu_2_Ti	Ag-rich	Cu(Ti,V)	Cu(Ti,V)	(Cu_x_Ni_1−x_)_2_Ti	Ag-rich	Cu(Ti,V)

**Table 5:** Chemical analyses in Figure 7.

at %	S	T	U	V	W	X	Y
Ag	1.4	3.0	90.3	5.1	3.0	-	0.4
Al	2.1	1.1	0.6	3.1	3.2	2.0	2.3
Cr	1.4	0.4	-	0.3	2.5	0.5	3.0
Cu	26.8	27.1	2.6	19.4	15.3	0.1	1.3
Ni	16.9	5.5	1.9	6.3	4.7	30.5	10.2
Sn	0.2	0.4	0.2	1.0	1.3	0.6	1.4
Ti	47.4	61.3	4.5	56.2	57.2	63.1	64.9
V	3.7	1.2	-	8.6	12.7	3.1	26.5
phase	CuNiTi	CuTi_2_	Ag-rich	CuTi_2_	CuTi_2_	Ti_2_Ni	Ti-rich

**Table 6:** Average shear strengths of brazed joints.

Filler Foil	Brazing Temperature	Brazing Period	Shear Strength
BAg-8	800 °C	300 s	172 ± 32 MPa
	850 °C	300 s	197 ± 35 MPa
Ticusil^®^	900 °C	300 s	220 ± 5 MPa
	950 °C	300 s	230 ± 44 MPa
